# Identification of the Efficient Enhancer Elements in FVIII-Padua for Gene Therapy Study of Hemophilia A

**DOI:** 10.3390/ijms25073635

**Published:** 2024-03-24

**Authors:** Rou Xiao, Yan Chen, Zhiqing Hu, Qiyu Tang, Peiyun Wang, Miaojin Zhou, Lingqian Wu, Desheng Liang

**Affiliations:** Center for Medical Genetics, School of Life Sciences, Central South University, Changsha 410078, China; xiaorou@sklmg.edu.cn (R.X.); chenyan@sklmg.edu.cn (Y.C.); huzhiqing@sklmg.edu.cn (Z.H.); zhoumiaojin@sklmg.edu.cn (M.Z.);

**Keywords:** Hemophilia A, FVIII-Padua enhancer C400, universal targeted gene modification, gene therapy, cell therapy

## Abstract

Hemophilia A (HA) is a common X-linked recessive hereditary bleeding disorder. Coagulation factor VIII (FVIII) is insufficient in patients with HA due to the mutations in the *F8* gene. The restoration of plasma levels of FVIII via both recombinant B-domain-deleted FVIII (BDD-FVIII) and B-domain-deleted *F8* (*BDDF8*) transgenes was proven to be helpful. FVIII-Padua is a 23.4 kb tandem repeat mutation in the *F8* associated with a high *F8* gene expression and thrombogenesis. Here we screened a core enhancer element in FVIII-Padua for improving the *F8* expression. In detail, we identified a 400 bp efficient enhancer element, C400, in FVIII-Padua for the first time. The core enhancer C400 extensively improved the transcription of *BDDF8* driven by human elongation factor-1 alpha in HepG2, HeLa, HEK-293T and induced pluripotent stem cells (iPSCs) with different genetic backgrounds, as well as iPSCs-derived endothelial progenitor cells (iEPCs) and iPSCs-derived mesenchymal stem cells (iMSCs). The expression of FVIII protein was increased by C400, especially in iEPCs. Our research provides a novel molecular target to enhance expression of FVIII protein, which has scientific value and application prospects in both viral and nonviral HA gene therapy strategies.

## 1. Introduction

Hemophilia A (HA) is an X-linked recessive hereditary bleeding disorder with a prevalence of approximately 1 in 5000 male neonates. At the end of 2022, WFH (World Federation of Hemophilia) released the latest global annual report showing that the number of confirmed hemophilia patients worldwide was 233,577 cases, of which 185,318 cases (nearly 80%) were HA patients. HA is caused by a deficiency in coagulation factor VIII (FVIII) owing to the mutations in *F8*. Severe patients, who account for more than 50% of HA cases, have only 1% or less of normal plasma FVIII activity and suffer from joint deformities and even disability due to repeated spontaneous bleeding [1]. The current clinical treatment of patients with HA is based on frequent infusions of FVIII replacement, which is expensive, and approximately 25–30% of cases develop inhibitors, leading to ineffective treatment [2,3].

HA is a monogenic genetic disorder with a clear pathogenic mechanism. Gene therapy has been regarded as one of the most promising strategies for HA. According to the Human Gene Mutation Database (HGMD), 4405 types of *F8* mutations cause HA (https://www.hgmd.cf.ac.uk/ac/gene.php?gene=F8 (accessed on 23 March 2024)). Based on the ectopic addition of *F8* genes, this strategy offers a universal treatment approach for patients with HA with different pathogenic mutation types. The *F8* cDNA whose length is about 7 kb poses a considerable challenge to current vector systems. Clinical treatment using recombinant B-domain-deleted FVIII (BDD-FVIII) has shown certain therapeutic effects, whereas the final activated form of FVIII does not contain the B domain [4].

Owing to the unlimited proliferative ability and multi-directional differentiation potential, induced pluripotent stem cells (iPSCs) have become the ideal targets for gene modification and the infinite source of transplantable cells [5,6]. Based on gene editing or gene addition to specific safe sites in vitro and subsequent screening for gene expression and specific integration, genetically modified cells can be further transplanted into the patients for treatment [7,8,9,10].

Efficient and stable expression of FVIII is one of the ultimate goals of gene therapy for HA, whether based on viral or nonviral strategies. In 2021, a study reported the co-segregation of a 23.4 kb tandem repeat in the proximal portion of the *F8* (encompassing the *F8* promoter, exon 1, and most of intron 1) with high FVIII activity (>400%) in two Italian families with severe thrombogenesis, and the mutation was named FVIII-Padua [11]. FVIII-Padua carriers in both families showed more than a 2-fold upregulation of *F8* mRNA levels, possibly related to open chromatin and enhancer elements within the repeat. The study detected these sequences using a luciferase reporter gene system and found that a 927 bp region (named C) and an 1880 bp region (named E) were associated with 45-fold and 15-fold increases, respectively, in the reporter gene expression in human umbilical vein endothelial cells. This discovery offers a potential avenue for the efficient expression of FVIII. Enhancers are non-coding cis-acting DNA elements near structural genes that activate or enhance gene transcription by interacting with transcription factors (TFs), cofactors, and chromatin complexes in the promoter region. Intron 1 of human genes has been widely reported to be involved in transcriptional regulation [12].

To screen a core enhancer element in FVIII-Padua to improve the *F8* expression, we screened the core elements in segments C and E of FVIII-Padua using a luciferase reporter gene system in various cell lines and identified an efficient enhancer element, C400. The B-domain-deleted *F8* (*BDDF8*) expression cassette driven by human elongation factor-1 alpha (EF1α) promoter in combination with C400 was integrated at the rDNA locus of patient-specific iPSCs with HA (HA-iPSCs) and normal human iPSCs (B6-iPSCs). The enhancing effect of C400 on the *F8* gene expression was confirmed in rDNA-specific integrated iPSCs, iPSC-derived endothelial progenitor cells (iEPC), and iPSC-derived mesenchymal stem cells (iMSC).

## 2. Results

### 2.1. Screening for Efficient Enhancer Elements in FVIII-Padua

**Figure 1:**
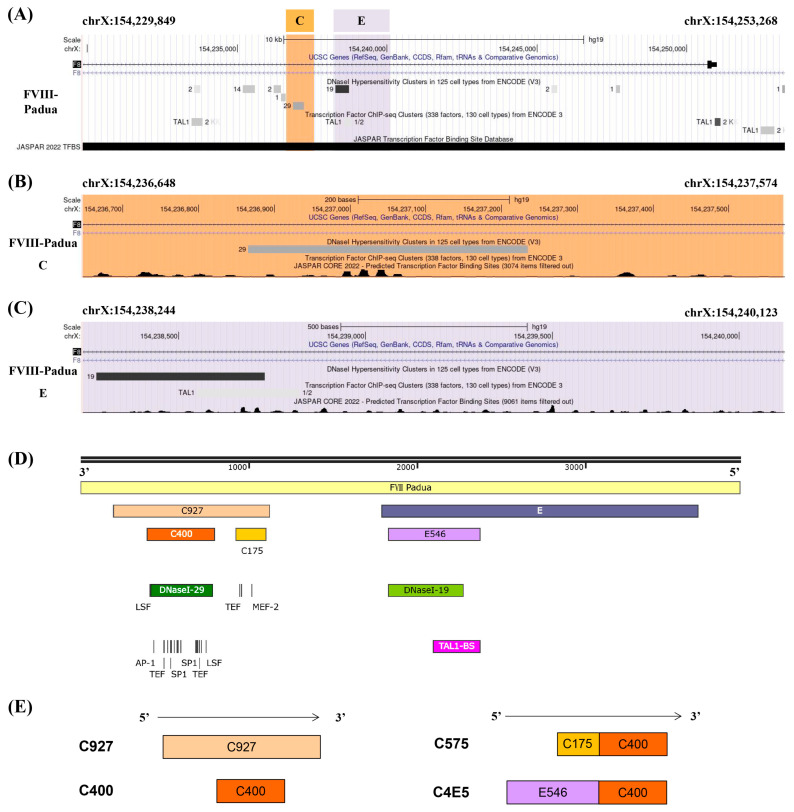
The candidate enhancers in FVIII-Padua. (**A**–**C**) Analysis of the gene position and functional domains in FVIII-Padua. The gene track of FVIII-Padua [11] in the genome browser and the gene position of putative enhancer region C and E are highlighted in orange and purple, and showing the DHSs and specific TF-binding sites from ENCODE and JASPAR. (**D**,**E**) Schematic diagram of the candidate enhancer elements in FVIII-Padua. A 4 kb sequence in FVIII-Padua containing fragments C and E is shown. Two TF-binding sites clusters, C400 and C175, exist in fragment C, and a DNase I-29 site is located on C400, which is active in 29 cell types. Fragment E contains a 546 bp region (E546) with a compound DNase I-19 site (active in 19 cell types) and a TAL1-binding site.

Several candidate core enhancer elements in FVIII-Padua were constructed based on the functional domain analysis of the gene sequence. Analysis of the sequence features of FVIII-Padua (Figure 1A) showed that both fragments E and C harbor type I deoxyribonuclease (DNase I) hypersensitive sites (DHSs) associated with DNA activity [13]; furthermore, binding sites for TFs are enriched in segment C (Figure 1B) and a TAL1 binding site is located in segment E (Figure 1C). Figure 1D shows the location relationship diagram of the candidate enhancer elements in a 4 kb sequence of FVIII-Padua containing C and E. Fragment C (referred to as C927 in this study) with a sequence length of 927 bp contained DHSs (DNase I-29). A 400 bp region named C400 spans the DNase I-29 and has a cluster of TF-binding sites (predicted by Cister, details are shown in Figure S1), such as Ets, which are related to physiological activities and *F8* expression of endothelial cells (ECs) [14,15]. In addition, a 175 bp region in C927 also has a cluster of TF-binding sites. This region was concatenated with C400 to form a 575 bp fragment named C575 (Figure 1E). Fragment E contains a DHS (DNase I-19) and a TAL1-binding site, which are adjacent and overlap in a small segment with a size of 546 bp (referred to as E546 in this study). To test whether these elements have additive effects, we concatenated E546 with C400 to form C4E5 (Figure 1E). The amplification of each element (sequences are shown in Table S1) was performed using PGL primers (Table S2).

**Figure 2:**
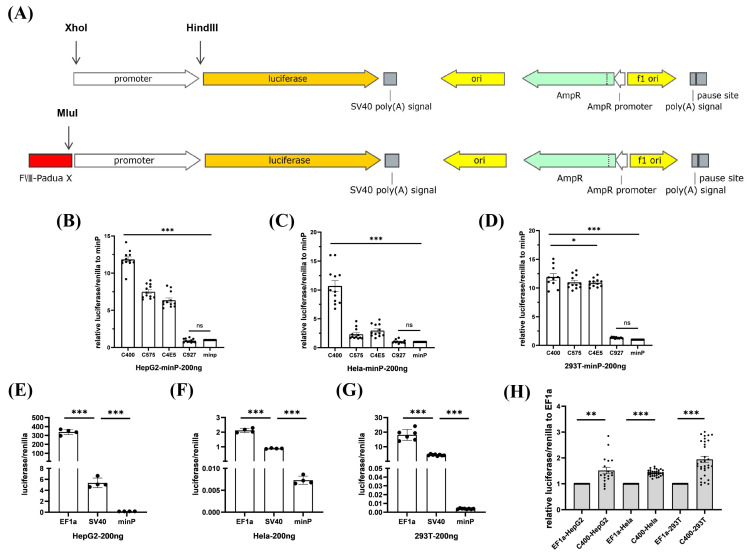
Screening of efficient enhancer elements in FVIII-Padua. (**A**) Schematic diagram of the luciferase gene reporter plasmid used for promoter and FVIII-Padua enhancer element screening, which has Amp resistance. The promoter is located upstream of the firefly luciferase cassette and can be replaced between the XhoI and HindIII restriction sites, and the enhancer element is inserted upstream of the promoter at the MluI cleavage site. (**B**–**D**) Relative expression of luciferase in HepG2, HeLa, and HEK-293T cells after transfection with minP-luciferase reporter plasmids with different putative enhancer elements, with Renilla luciferase expression as an internal control. *** *p* < 0.001, * *p* < 0.05, ns represents no significant difference, and the bars represent mean ± SEM. (**E**–**G**) Luciferase expression in HepG2, HeLa, and HEK-293T cells after transfection with luciferase reporter plasmids driven by different promoters, with Renilla luciferase expression as an internal control. *** *p* < 0.001, and the bars represent mean ± SEM. (**H**) Relative luciferase expression in HepG2, HeLa, and HEK-293T cells after transfection with C400-EF1α-pGL3, with Renilla luciferase expression as an internal control. *** *p* < 0.001, ** *p* < 0.01, and the bars represent mean ± SEM.

To screen the core enhancer elements in FVIII-Padua, the putative enhancer elements (C400, C575, C4E5, and C927) were respectively cloned into a minP-pGL3 vector, a luciferase reporter gene system driven by the minP promoter. Moreover, pGL3-Promoter Vector (GenBank Accession Number U47298) offered the vector backbone (Figure 2A), whose original promoter was SV40 and could be replaced by XhoI and HindIII digestion. After amplification of the minP promoter using minP-pGL3-F/R primers (Table S1), it was ligated into the reporter vector. A putative enhancer element was inserted into the MluⅠ enzyme cutting site. Sanger sequencing confirmed the successful construction of luciferase gene reporter plasmids with different elements inserted upstream of the minP promoter (Figure S2A). Co-transfection was performed with the internal control plasmid pRL-TK vector (GenBank Accession Number AF025846) expressing Renilla luciferase. Gene expression was indicated by luminescence detection. The luciferase assay results (Figure 2B–D) showed that in all three cell lines (HepG2, HEK-293T, and HeLa), except for C927, the luciferase expression in all groups with added elements was significantly increased. Further, the enhancer effect of C400 was the most obvious, reaching more than 10 times that of the group with only the minP promoter. C575 and C4E5 also showed enhancer effects, especially in the HEK-293T cell line.

In previous studies, the EF1α promoter has been shown to drive high expression of exogenous genes in various cells. They can maintain stability during stem cell differentiation [16,17]. In this study, we constructed the EF1α-pGL3 vector, a luciferase gene reporter plasmid driven by the EF1α promoter (Figure S2B). EF1α was significantly superior to SV40 and minP, considering the regulation ability for luciferase expression in all three cell lines at the same transfection dose (Figure 2E–G). Subsequently, we inserted the C400 upstream of the EF1α in the EF1α-pGL3 vector (Figure S2C). The expression of luciferase driven by EF1α was improved (150–200%) by C400 in all three cell lines (Figure 2H). The C400 element ultimately achieved significant enhancement of gene expression through its synergistic association with EF1α.

### 2.2. Efficient Enhancer C400 in FVIII-Padua Improved F8 Expression Driven by EF1α

**Figure 3:**
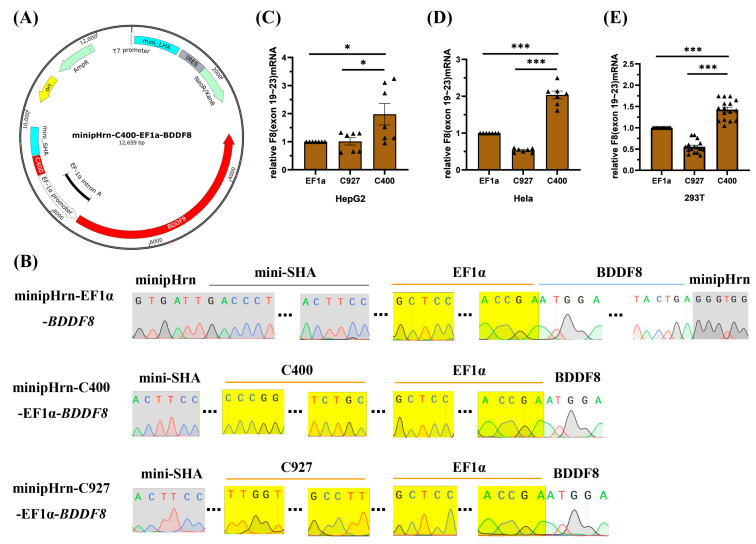
Construction and transfection of *BDDF8* plasmids. (**A**) Map of the minipHrn-C400-EF1α-*BDDF8* vector, with the C400 element located upstream of the EF1α-driving *BDDF8*, contains a *NEO* and an *Amp* cassette. (**B**) Sequencing results of the *BDDF8* expression plasmids, with the yellow background indicating the sequences of EF1α promoter, C400 or C927 element that are perfectly matched with the theoretical sequences, the gray background indicating the vector backbone, and the white background indicating the *BDDF8* sequence (only the connecting region is shown). (**C**–**E**) Relative transcription levels of *F8* (exons 19–23) after transfection into HepG2, Hela, and HEK-293T cells are detected using RT-qPCR with GAPDH as the internal control. *** *p* < 0.001, * *p* < 0.05, and the bars represent mean ± SEM.

To further investigate whether the C400 element could enhance the expression of *F8* driven by EF1α, we constructed a *BDDF8* expression cassette, located on a plasmid targeting the human rDNA locus (Figure 3A), and the plasmid backbone was constructed previously [18]. Sequencing results showed that we successfully constructed the *F8* expression plasmids (Figure 3B), minipHrn-EF1α-*BDDF8*, minipHrn-C400-EF1α-*BDDF8*, and minipHrn-C927-EF1α-*BDDF8* (for further verification). Subsequently, we separately transfected these three plasmids into cell lines, and the results of *F8* transcription level showed that the C400 element could significantly enhance *F8* expression driven by EF1α in all three cell lines (1.5–2-fold increase), whereas C927 did not exhibit any enhancing effects (Figure 3C–E), which was consistent with the luciferase assays.

### 2.3. rDNA-Specific Integration of the Novel F8 Cassette into iPSCs of Different Genetic Backgrounds

**Figure 4:**
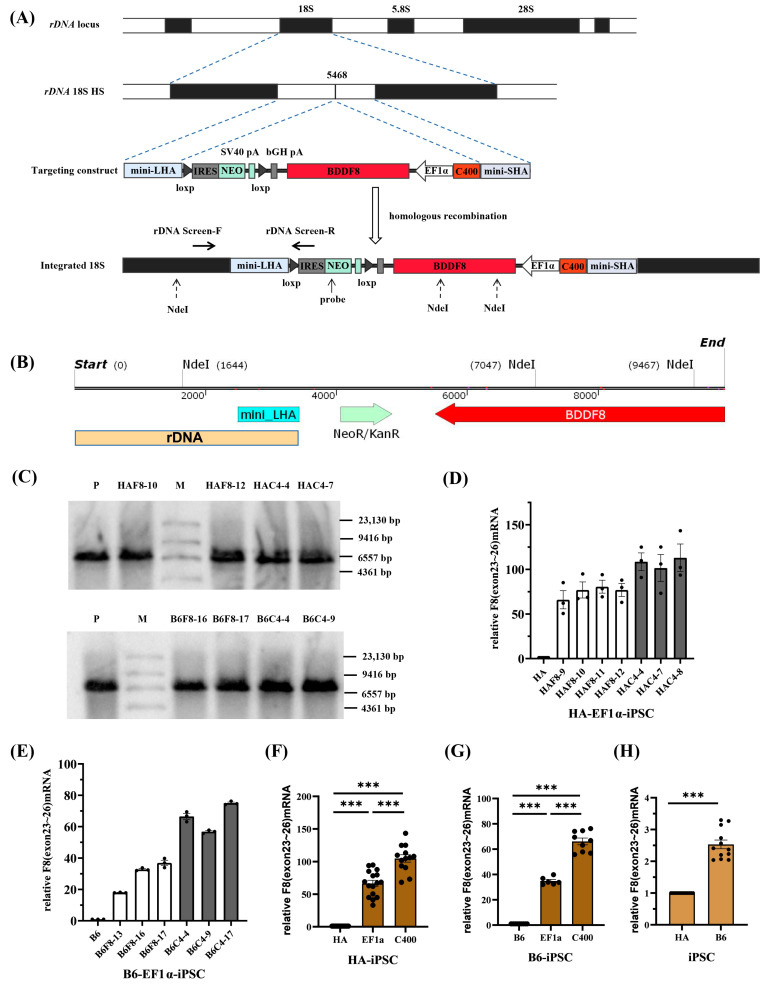
rDNA-specific integration of novel *F8* expression cassette into iPSCs of different genetic backgrounds. (**A**) A schematic of the rDNA locus and rDNA-specific integration of efficient *F8* expression cassette at the 5468 site within the rDNA locus. (**B**) The distribution of the NdeI sites after the integration of the *BDDF8* gene at the 5468 site. (**C**) Southern blotting results for the iPSCs with targeted integration of the *BDDF8* gene at the rDNA locus showing the specific bands of 5.4 kb or 7.8 kb for the integration event (M, DIG-labeled Molecular WeightII). (**D**,**E**) Relative transcription levels of *F8* (exons 23–26) in the HA-iPSCs or B6-iPSCs were detected using RT-qPCR, with GAPDH as the internal control. The white columns represent targeted clones with EF1α-*BDDF8*, and the gray columns represent targeted clones with C400-EF1α-*BDDF8*. (**F**,**G**) Statistic analysis of the relative transcription levels of *F8* (exons 23–26). GAPDH is used as the internal control. (**H**) Relative transcription levels of *F8* (exons 23–26) in B6-iPSCs to HA-iPSCs. GAPDH is used as the internal control. *** *p* < 0.001, and the bars represent the mean values ± SEM.

rDNA locus is a safe and effective ideal site for gene targeting. In rapidly proliferating stem cells (such as iPSCs), transcription in the rDNA locus is particularly active; at the rDNA locus, the chromatin is loose and prone to homologous recombination (HR)-mediated targeting [19,20]. Our team previously developed and optimized a novel nonviral targeting vector: minipHrneo [21], which uses a portion of the rDNA sequence as a homologous guiding sequence to integrate an exogenous therapeutic gene into the rDNA locus through HR (Figure 4A). Using the donor vector minipHrneo with the help of TALENickases plasmids [22] (TALEN L and TALENickase R D450A), we have achieved an efficient targeted integration of various exogenous genes, including *BDDF8*, *F9*, *miniDystrophin*, *IL-24*, and *TRAIL* into the rDNA locus. The stable expression of exogenous genes in vivo and in vitro were confirmed [18,21,23,24,25].

To explore the enhancer effect of C400 and its application for universal targeted gene therapy for HA in different genetic backgrounds, we nucleofected *F8* expression vectors into the rDNA loci of HA-iPSCs and normal human iPSCs (B6-iPSCs), respectively. Both iPSCs were previously established [26,27]. After selection using G418, single clones were selected for identification. PCR screening was performed with primers rDNA-screen-F/R (Table S1) spanning the mini-long homologous arm. Gel electrophoresis results showed that more than 80% of the clones were positive for the expected band of 1.6 kb (Figure S3A–D, left), which were consistent with the theoretical sequences (Figure S3A–D, right). The successfully targeted cells with EF1α-*BDDF8* were named HAF8 and B6F8, and different clones were designated with clone numbers (e.g., the tenth clone of HAF8 was named HAF8-10). Similarly, cells integrated with C400-EF1α-*BDDF8* were named HAC4 and B6C4.

To further confirm the rDNA-specific integration of exogenous genes, we selected two clones from each of the four integrated iPSCs for Southern blotting. Theoretically, after complete digestion with NdeI, the gDNA of rDNA-specific integrated clones can hybridize with probes homologous to the *NEO* gene and exhibit a 5.4 kb band. If the digestion was incomplete, a 7.8 kb band may also be observed (Figure 4B). Southern blotting confirmed that eight targeted clones were positive for the expected bands (Figure 4C).

Because of an inversion in intron 22 of the *F8* in the HA-iPSCs, we validated the mRNA transcript of the exogenous *F8* gene through PCR using primers *F8* RT-19F/23R spanning exons 19–23 (Table S1). All positive clones of HAF8-iPSCs and HAC4-iPSCs, as well as normal human B6-iPSCs, had transcripts with correct splicing of exons 19–23 (Figure S3E,F, left). These were sequenced and verified to be consistent with the theoretical sequence (Figure S3E,F, right). When the *F8* intron 22 inversion occurs, the exons 23–26 will be spliced into exon 1 of an unrelated gene, which will encode a 2.6 kb transcript and the transcription level is equivalent to the normal level [28]. And in order to observe the gene transcription ability of HA itself, we used the exons 23–26 amplification.

We then randomly selected positive clones and performed RT-qPCR using the *F8* RT-23F/26R primers, spanning the exons 23–26 of *F8*. The results showed that C400 significantly enhanced the ability of EF1α to drive *F8* transcription in HA-iPSCs (Figure 4D) and B6-iPSCs (Figure 4E). Statistical analysis showed that the *F8* transcription level in HAF8-iPSCs was approximately 60 times that in HA-iPSCs and over 100 times that in HAC4-iPSCs (Figure 4F). Compared with the transcription level of *F8* in B6-iPSCs, that in B6F8-iPSCs and B6C4-iPSCs were approximately 35 and 70 times higher, respectively (Figure 4G). For different genetic backgrounds, the *F8* transcription level in B6-iPSCs was approximately 2.5 times that in HA-iPSCs (Figure 4H).

Before proceeding to the next research step, we performed safety (Figures S4 and S5) and multipotency assays (Figure S6) on the four sets of rDNA-specific integrated clones. The sequencing results for the three most likely off-target sites predicted by the website (Table S3) showed no detectable off-target events. Karyotype analysis showed that the chromosome compositions of HAF8-10, HAC4-4, B6F8-16, and B6C4-9 were consistent with those of HA-iPSCs and B6-iPSCs (46, XY), with no abnormalities.

Immunofluorescence showed that all integrated iPSCs expressed the pluripotency markers OCT4, SOX2, SSEA4, and TRA-1-60 (Figure S6A). Teratoma formation experiments confirmed that all iPSCs had the potential to differentiate into the three germ layers in vivo (Figure S6B).

We have successfully constructed iPSC cells with rDNA-specific integration of EF1α-*BDDF8* or C400-EF1α-*BDDF8*, which can be used for research and application of HA gene therapy. We also demonstrated that the core efficient enhancer element C400 in FVIII-Padua improved the expression of *F8* in different genetic backgrounds.

### 2.4. rDNA-Specific Integrated iEPCs and the Expression of F8

**Figure 5:**
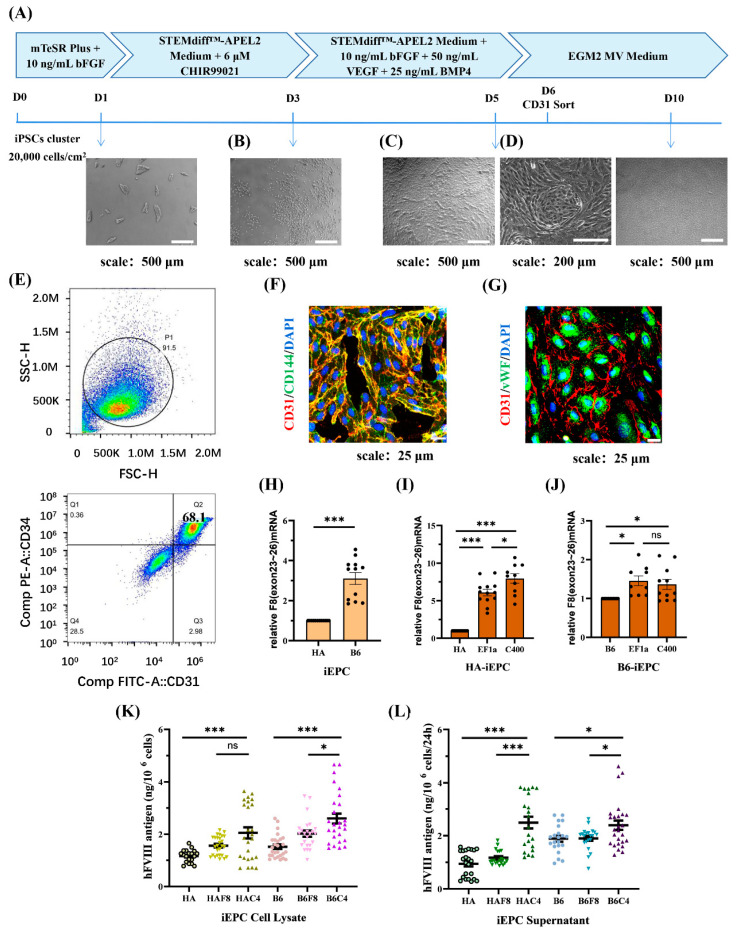
rDNA-specific integrated iEPCs and the expression of *F8*. (**A**) Schematic diagram of the iEPCs differentiation process, which involves a 5 d small molecule induction followed by maintenance in endothelial growth medium. (**B**–**D**) Cell morphological changes during the differentiation. (**E**) The differentiation efficiency of CD31 and CD34 double positive iEPCs is analyzed using flow cytometry. (**F**) Immunofluorescence of endothelial progenitor cell markers CD31 (red) and CD144 (green), and DAPI (blue) staining the nuclei. (**G**) Immunofluorescence of endothelial markers CD31 (red) and vWF (green) and DAPI (blue) staining the nuclei. (**H**) Relative transcription levels of *F8* (exons 23–26) in B6-iEPCs to HA-iEPCs. GAPDH is used as the internal control. (**I**,**J**) Analysis of the relative transcription levels of *F8* (exons 23–26) in the HA-iEPCs and B6-iEPCs. GAPDH is used as the internal control. (**K**) ELISA results for the FVIII antigen levels in the cell lysate of iEPCs. (**L**) ELISA results for the FVIII antigen levels in the cell culture supernatant of iEPCs. *** *p* < 0.001, * *p* < 0.05, “ns” means no significant difference. The bars represent the mean values ± SEM.

Kumaran et al. [29] and Gage et al. [30] confirmed that liver sinusoidal endothelial cells (LSECs) were the main cell type synthesizing and secreting FVIII. FVIII is structurally unstable in the plasma and is easily degraded by proteolytic enzymes. ECs can produce vWF to form a complex with FVIII, thereby maintaining stability [31,32]. In this study, we followed a previously reported differentiation protocol for producing ECs from iPSCs [33]. After 6 d, typical epithelial-like cells were obtained (Figure 5A).

During the differentiation process, cell aggregates were dispersed into individual irregularly shaped cells after 2 d of induction in an APEL2 medium containing 6 μM CHIR99021 (Figure 5B). After replacing CHIR99021 with VEGF, BMP4, and bFGF, the cells proliferated rapidly and became regular and spindle-shaped. At a high density, the cells connected to form arches and circles, which were similar to blood vessels (Figure 5C,D).

On day 6, flow cytometry analysis with the EPC markers CD31 and CD34, was performed and showed a differentiation efficiency of 68.1% (Figure 5E). Furthermore, iEPCs were sorted using CD31 magnetic beads and maintained in ECGM-MV2 medium in a Matrigel-coated plate. The immunofluorescence results showed that cells cultured for 3 d after CD31 sorting expressed the EPC markers CD31 and CD144, and both markers co-localized on the cell membrane (Figure 5F). After 8 d, the cells expanded in size and expressed the endothelial markers CD31 and vWF, with vWF being largely present in the cytoplasm (Figure 5G).

We detected the expression of *F8* at the iEPCs stage. The transcription level of *F8* in B6-iEPCs was approximately three times of that in HA-iEPCs (Figure 5H). The *F8* transcription level in HAF8-iEPCs and in HAC4-iEPCs was six times and eight times of that in HA-iEPCs, respectively (Figure 5I). In B6, the transcription level of *F8* in B6F8-iEPCs was 1.5 times that in B6-iEPCs (Figure 5J). Although the use of the C400 element did not further enhance *F8* transcription in B6F8-iEPCs, ELISA results showed that the synthesis (Figure 5K) and secretion (Figure 5L) of FVIII protein in B6C4-iEPCs was significantly increased. We detected ~2.5 ng of FVIII protein in the cell culture supernatants of 10^6^ HAC4-iEPCs and B6C4-iEPCs after 24 h, which was significantly higher than those in the other groups (Figure 5L). The 2.6 kb transcript in HA-iEPCs encoded a truncated and inactive FVIII protein that is incapable of secretion, therefore, the detected FⅧ expression level is equivalent to the normal level in the cell lysate while significantly lower in the supernatant [28]. The truncated FVIII protein can be detected by the FⅧ C-terminal antibody used in ELISA.

### 2.5. rDNA-Specific Integrated iMSCs and the Expression of F8

**Figure 6:**
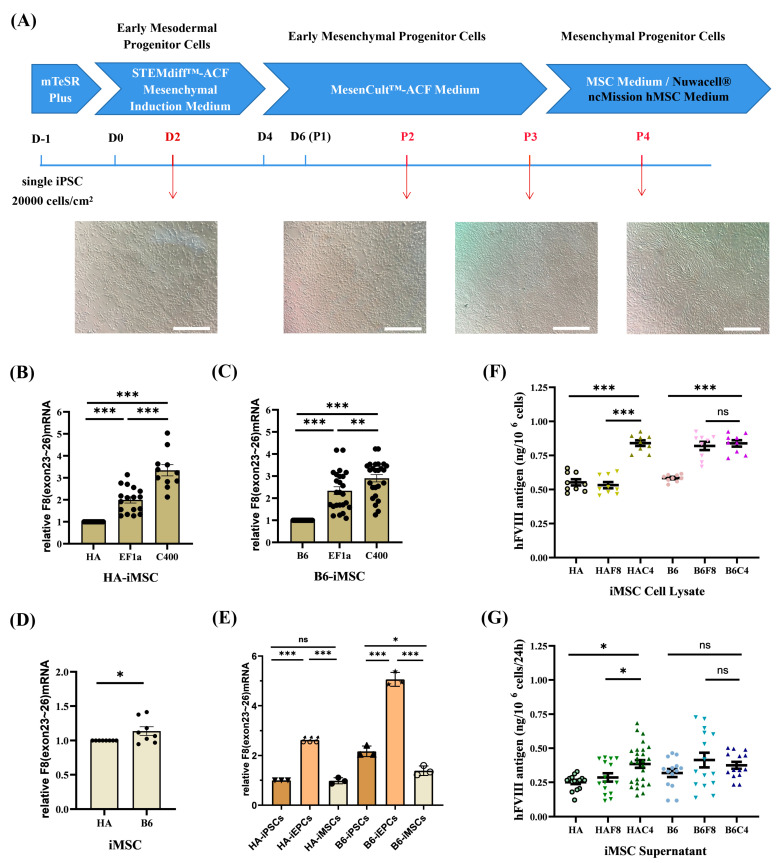
rDNA-specific integrated iMSCs and the expression of *F8*. (**A**) Schematic diagram and cell morphology changes of the iMSCs differentiation process. Scale bar: 200 μm. (**B**,**C**) Analysis of the relative transcription levels of *F8* (exons 23–26) in B6-iMSCs and HA-iMSCs. GAPDH is used as the internal control. (**D**) Relative transcription levels of *F8* (exons 23–26) in B6-iMSCs to HA-iMSCs. GAPDH is used as the internal control. (**E**) Comparison of the relative transcription levels of *F8* (exons 23–26) at various cell stages without gene modification (HA/B6-iPSCs, iMSCs and iEPCs). GAPDH is used as the internal control. (**F**) ELISA results for the FVIII antigen levels in the cell lysate of iMSCs. (**G**) ELISA results for the FVIII antigen levels in the cell culture supernatant of iMSCs. *** *p* < 0.001, ** *p* < 0.01, * *p* < 0.05, “ns” means no significant difference. The bars represent the mean values ± SEM.

MSCs are a type of stem cell widely used in cell therapy. They are considered ideal therapeutic cells for many diseases owing to their low immunogenicity, strong proliferative ability, wide and easy-to-obtain tissue sources, multi-directional differentiation potential, and homing and paracrine effect [34,35,36,37,38]. Under physiological conditions, MSCs synthesize and secrete FVIII [39] and differentiate into ECs [40]. Co-transplantation of MSCs and ECs resulting in enhanced cell engraftment and FVIII expression in mice [41,42]. Thus, MSCs can be effective for HA gene therapy. In this study, iPSCs were differentiated into MSCs using the STEMCELL Mesenchymal Progenitor Kit (Figure 6A). After approximately 21 d of differentiation, we obtained iMSCs with typical fibroblast-like morphology and good proliferative capacity. Flow cytometric analysis (Figure S7) showed that the surface markers CD44, CD72, CD90, and CD105 were positive in all iMSCs, whereas CD45, CD34, and HLA-DR were negative, which was consistent with the characteristics of MSCs. In addition, all the iMSCs had the potential to differentiate into adipocytes (left), osteoblasts (middle), and chondrocytes (right) (Figure S8).

At the iMSC stage, the transcription level of *F8* in HAF8-iMSCs was 2 times that in HA-iMSCs, whereas in HAC4-iMSCs, it further increased by over 3 times that in HA-iMSCs (Figure 6B). The *F8* transcription level of B6F8-iMSCs was nearly 2.5 times that of B6-iMSCs, and using the C400 element further increased it to 3 times that of B6-iMSCs (Figure 6C). Unlike iPSCs and iEPCs, there was little difference in the transcript levels of *F8* between unmodified B6-iMSCs and HA-iMSCs (Figure 6D). We also found that the *F8* mRNA in unmodified iEPCs was approximately 2.5–3 times that in iPSCs and iMSCs (Figure 6E).

ELISA results showed that the synthesis and secretion of FVIII in HAC4-iMSCs were significantly higher than in HAF8-iMSCs (Figure 6F,G). In the B6 background, targeted integration of EF1α-*BDDF8* improved the expression of FVIII protein (Figure 6F). However, the significant increase in the transcription level of *F8* in B6C4-iMSCs did not directly transform to an equal increase in FVIII protein. FVIII synthesized by MSCs is distributed around the nucleus rather than being stored in granules, indicating that MSCs have independent regulatory mechanisms for FVIII synthesis and secretion that are distinct from those in ECs [43]. However, these mechanisms have not been fully studied. The C400 element originates from the *F8* and may have different effects on regulating FVIII expression in EPCs and MSCs; this requires further study.

## 3. Discussion

FIX-Padua produces high levels of FIX activity compared with normal FIX [44,45,46,47]. Currently, FIX-Padua is the standard transgene used for HB gene therapy [48,49,50]. Unlike FIX-Padua, the FVIII-Padua mutation leads to increased FVIII expression [11]. To find out the core enhancer elements of FVIII-Padua that enhance FVIII expression, we screened the elements in FVIII-Padua and identified an enhancer, C400, for the first time. C400 is only 400 bp in length but can efficiently enhance gene expression, including *F8*, in various cell lines and stem cells of different genetic backgrounds. This finding has scientific value and application prospects for viral and nonviral HA gene therapies.

DHSs on the C400 element are markers of active chromatin [51], and are accessible to DNA polymerases for recognition, binding, and cutting, which promotes gene transcription and expression. Currently, the DHSs-related element commonly used to enhance gene expression is HS-40, located 40 kb upstream of the ζ2 gene encoding globin alpha chains, which are erythroid-specific [52]. The DHSs on the enhancer element C400 identified in this study were widely active in 29 different cell types, making them widely applicable in mammalian cell lines. In HepG2, HeLa, iPSC, and EC, which were included in the 29 active cell types according to the UCSC database; C400 but not E546 element showed consistent enhancement effects. Therefore, the mechanisms associated with these DHSs should be further explored.

In this study, no other candidate elements containing C400 were found to have enhanced effects compared with C400 in independent transfection experiments in each of the three cell lines. In eukaryotic cells, the chromatin complex structure exists in a supercoiled form. Even though the enhancer and regulated genes are separated by a long nucleotide sequence, they are close in space, which enables the enhancer to bind to TFs and RNA polymerase II. However, longer sequences may form more complex structures and bind to different regulatory proteins [53]. This may lead to spatial hindrance and transcriptional blockage, especially when the C400 element is adjacent to the promoter, which may cause repression of the multi-segment combination. However, the exact underlying mechanism requires further investigation. Smaller enhancer elements in C400 are yet to be discovered. Technologies and databases that study gene expression regulation at different levels provide powerful tools for mechanistic research [51,54,55,56,57,58,59,60].

Currently, the main clinical research on gene therapy for HA involves the introduction of the therapeutic gene *BDDF8* into liver cells. The Food and Drug Administration (FDA) recently approved an AAV gene therapy product (Roctavian) for treating HA. However, antibodies against AAV lead to inaccessibility of treatment in some populations and the impossibility of secondary treatment, and the loss of viral vectors and transgenes also make AAV strategies unsuitable for the early treatment window [61,62,63]. Although the cell therapy strategy is still in the stage of proving efficacy, it offers the possibility to cover all HA patients and treatment windows. With the rapid development and maturity of stem cell reprogramming technology, cell therapeutic strategies based on gene-modified iPSCs have been widely studied for various diseases [64,65,66,67]. In the case of cell therapy, the potential risks of tumorigenesis and unintended differentiation must be controlled by strengthening the uniform quality of the products, visual tracking, strict long-term supervision, and other means [68]. In addition, the results of this study suggested that EPCs may be superior to MSCs as FVIII-expressing cells. LSECs are the main cell type that produce FVIII, and the vWF generated in ECs can bind to FVIII to form a stable complex, prolonging their half-life of FVIII and reducing immunogenicity [69]. Therefore, EPCs are important target cells for HA gene therapy. It seems likely that the targeted disruption of HLA genes or knockout of *B2M* genes can reduce cell immunogenicity and solve the immune problems associated with EPCs/ECs transplantation [70,71].

Our study identified a novel target, C400, for enhancing the expression of *F8*. Based on a strong promoter, C400 can still significantly enhance the transcription of *F8* in HEK-293T cells and iPSCs; and the HEK-293 cell line is one of the main producers for recombinant FVIII protein [72]. Although we improved the *F8* transcription level by C400, the FVIII protein expression was not robust, in which the limited ability of translation may be related to the coding sequence and protein structure of *BDDF8*. Lower levels of protein translation may make the differences more difficult to observe. And by codon or structure optimization of *BDDF8*, the expression of FⅧ could be further improved. A study has shown that in humanized liver (human–liver chimeric FRG-KO) model mice, the average expression level of hybrid human/pig FVIII molecules (91% human and 9% pig) delivered by adeno-associated virus (AAV) was as high as 480% of the normal levels [73]. Combined with the C400 enhancer element, optimization strategies for FVIII coding sequences and protein structures have the potential to further upregulate *F8* expression in both viral and nonviral HA gene therapies. In addition, the transcriptional level of *F8* in B6C4-iEPCs was equal to that in B6F8-iEPCs. However, the expression of FVIII was significantly increased. The presence of predictable transcription factor binding sites on C400 elements related to endothelial and vascular physiological activities may mediate the translation and expression of FVIII [14,15,74], which should be further confirmed. Since the C400 element comes from the *F8* and LSECs are physiological cells that synthesize and secrete FVIII, the enhancer C400 can be further explored with the *F8* gene driven by the *F8* promoter or EC-specific promoter.

## 4. Materials and Methods

### 4.1. Plasmids Construction

Construction of luciferase gene reporter plasmids used high-fidelity LA Taq DNA Polymerase (Takara Biomedical Technology, Beijing, China); the promoters and elements were amplified using homologous recombination primers. The fragments were ligated to the vector backbone using the ClonExpress II One Step Cloning Kit (Vazyme, Nanjing, China), respectively. An additional step of fragments connection was required for C575 and C4E5. pGL3 (Promega, Madison, WI, USA) was used as the vector backbone. The pRL-TK vector (Promega) expressing Renilla luciferase was used as an internal control.

Construction of *F8* plasmids targeting the rDNA locus used the minipHrn-*2bF8* plasmid previously constructed [18] as the backbone, which contained *BDDF8* sequence. The original promoter was removed through digestion with BamHI (New England BioLabs, Hitchin, UK). The sequence of *BDDF8* was completed through homologous recombination. NruI (New England BioLabs) and BsiWI (New England BioLabs) were used to insert the promoter and enhancer elements, respectively.

### 4.2. Cell Culture

All cells were routinely cultured (at 37 °C and 5% CO_2_) in a humidified incubator.

(1) Cell lines were purchased from Procell Life Science & Technology (Wuhan, China) and cultured in DMEM/HG (Gibco, Grand Island, NY, USA) supplemented with 10% fetal bovine serum (FBS; Gibco) and split every 3–4 days.
(2) iPSCs, HA-iPSCs [26], and B6-iPSCs (also referred to as hiPSCs) [27] have been generated. All iPSCs were cultured on Matrigel (BD Biosciences, Franklin Lakes, NJ, USA) -coated well plates (Corning, NY, USA) in mTesR plus medium (StemCell Technologies, Vancouver, BC, Canada).
(3) iEPCs derived from iPSCs were cultured on Matrigel-coated well plates in endothelial cell (EC) Growth Medium MV2 (Lonza, Basel, Switzerland).
(4) iMSCs derived from iPSCs were cultured on 0.1% gelatin- (STEMCELL Technologies) coated dishes (Corning) in MSC medium containing α-MEM basal medium (Thermo Fisher Scientific, Waltham, MA, USA) supplemented with 10% FBS (Gibco), 2 mM GlutaMAX (Gibco), and 0.1% basic fibroblast growth factor (bFGF; Gibco).

### 4.3. Cell Lines Transfection

Transfection of luciferase gene reporter plasmids: Cells were seeded at a density of 100,000 cells per well in a 24-well plate 18–24 h before the transfection. Luciferase gene reporter plasmid (200 ng) and pRL-TK (10 ng) were co-transfected into HepG2 cells using TurboFect Transfection Reagent (Thermo Fisher Scientific) according to the manufacturer’s protocols, and HeLa or HEK-293T cells were transfected using Lipofectamine 2000 (Thermo Fisher Scientific) according to the manufacturer’s instructions.

Transfection of *F8* plasmids: Cells were seeded at a density of 200,000 cells per well in a 12-well plate 18–24 h before the transfection. *F8* plasmids (300 ng) were transfected into HepG2 cells using TurboFect Transfection Reagent; and HeLa or HEK-293T cells were transfected using Lipofectamine 2000.

The medium was changed 8–12 h after transfection, and the expression of luciferase or *F8* was detected after 48 h.

### 4.4. Luciferase Activity Assays

Luciferase assays were performed using the Dual-luciferase Reporter Assay (Promega) according to the manufacturer’s instructions, and the Firefly and Renilla luciferase activities were detected using a Sirius Single tube chemiluminescence detector (Berthold Technologies, Stuttgart, Germany). The transfections were independently replicated at least four times.

### 4.5. RT-PCR and RT-qPCR

Total RNA was isolated using the TRIzol reagent (Sigma-Aldrich, St. Louis, MO, USA), and cDNA was prepared by removing the residual gDNA and reverse transcribed with HiScript II Q RT SuperMix for qPCR (+gDNA wiper) (Vazyme) according to the manufacturer’s instructions.

For RT-qPCR, 1000 ng of the RNA sample was reverse transcribed and then the cDNA was diluted at a ratio of 1:2. All qPCR were performed using the Taq Pro Universal SYBR qPCR Master Mix (Vazyme) according to the manufacturer’s instructions.

### 4.6. Nucleofection and Characterization of iPSCs

In a previous study, we constructed a nonviral human rDNA locus-targeting vector: minipHrneo [22]. The iPSCs were nucleofected using the Human Stem Cell Nucleofector Kit 2 (Lonza) according to the manufacturer’s instructions. Briefly, iPSCs were dissociated into single cells using the TrypLE Express (Thermo Fisher Scientific). For 2 × 10^6^ cells, 4 µg of each TALENickases plasmid and 5 µg of *F8* plasmid were used. Then, the nucleofected cells were seeded on Matrigel-coated 6-well plates in mTesR Plus medium with 10 µM of Y27632. After 3 d, G418 (Sigma-Aldrich) was used for selection at a final concentration of 50 µg/mL. The cells were then dissociated into single cells, and 800 cells were seeded on Matrigel-coated 6 cm dishes and cultured in mTesR Plus medium containing 10% CloneR (STEMCELL Technologies).

After 9–11 d, clones were picked, expanded, and screened via PCR using the screen primers rDNA-screen-F/R. The off-target activities of TALENickases were analyzed via PCR and Sanger sequencing. Additionally, karyotypes were analyzed using G-banding, and the pluripotency was identified using immunofluorescence of stem cell markers (OCT4, SOX2, SSEA4, and TRA-1-60 (Cell Signaling Technology, Boston, MA, USA)) and H&E staining of teratoma.

### 4.7. Southern Blotting

First, 10 µg of genomic DNA was digested with NdeI (New England Biolabs) at 37 °C overnight, and each product was loaded on 1% agarose gel for electrophoresis at 150 V for 2 h. The bands in the gels were then transferred overnight to a positively charged nylon membrane with DIG-labeled Molecular Weight II as a DNA marker. Then, the blotted membrane was baked at 75 °C for 2 h. Subsequently, the membrane was hybridized with DIG Easy Hyb Granules and detected with CDP-Star according to the manufacturer’s instructions. Unless otherwise stated, all the reagents and materials were purchased from Roche (Basel, Switzerland).

### 4.8. Differentiation and Characterization of iEPCs and iECs from hiPSCs

Differentiation of iEPCs [33]: Briefly, iPSCs were manually seeded as small clusters onto Matrigel-coated 6-well plates in mTeSR plus supplemented with 10 ng/mL of bFGF (Thermo Fisher Scientific). After 1 d, the culture medium was switched with StemDiff APEL2 medium (STEMCELL Technologies) containing 6 μM of CHIR99021 (Selleck Chemicals, Houston, TX, USA) for 2 d. Subsequently, the cells were cultured in StemDiff APEL2 medium supplemented with 25 ng/mL bone morphogenetic protein 4 (BMP4; PeproTech, Cranbury, NJ, USA), 50 ng/mL vascular endothelial growth factor (VEGF; PeproTech), and 10 ng/mL bFGF2 for 2 d. After the 5-d induction, iEPCs were generated and sorted using a CD31 MicroBead Kit (Miltenyi Biotec, Cologne, Germany) for further generation of iECs in ECGM-MV2. The cells were characterized using flow cytometry and an immunofluorescence assay.

Flow cytometry: Cultured cells were dissociated with Accutase (Innovative Life Technologies, San Diego, CA, USA) and filtered using a 40 μm nylon mesh (Corning). Then, the cells were washed in 500 µL Dulbecco’s phosphate-buffered saline (DPBS) with 5% FBS (Gibco), followed by incubation in 100 µL 5% FBS with antibodies PE mouse anti human CD34 (BD Biosciences) and FITC mouse anti human CD31 (BD Biosciences) at 4 °C for 30 min in the dark. Finally, the stained cells were washed twice with DPBS and resuspended in 200 µL DPBS for flow cytometry (CYTEK Biosciences, Fremont, CA, USA) detection.

Immunofluorescence: The cells seeded on chamber slides were fixed in 4% paraformaldehyde for 15 min and permeabilized in PBST (DPBS containing 0.1% Triton X-100 (Sigma-Aldrich)) for 18 min. The cells were then blocked in 5% bovine serum albumin (BSA; R&D Systems, Minneapolis, MN, USA) for 30 min and then incubated with primary antibodies, namely monoclonal anti-CD31 (PECAM-1), antibody produced in mouse (Sigma-Aldrich), anti-VE cadherin antibody, intercellular junction marker (Abcam, Cambridge, UK), and anti-von willebrand factor (vWF) antibody (Abcam) for 1 h at room temperature. After thorough washing, the cells were blocked with 5% BSA for 30 min and incubated with the secondary antibodies (BD Biosciences) for 1 h at room temperature in the dark. Nuclei were stained with DAPI (Sigma-Aldrich) for 8 min. Images were captured using a confocal microscope (Leica, Wetzlar, Germany).

### 4.9. Differentiation and Characterization of iMSCs from iPSCs

iMSCs were differentiated from iPSCs using the STEMdiff mesenchymal progenitor kit (STEMCELL Technologies) according to the manufacturer’s protocols. Briefly, iPSCs were seeded in mTesR Plus medium containing 10 µM Y27632 for 2 d. The medium was then switched to STEMdiff-ACF mesenchymal induction medium (STEMCELL Technologies) for 3 d. Subsequently, the cells were cultured in STEMdiff-ACF medium (STEMCELL Technologies) for an additional 2 d and then passaged into 6-well plates pre-coated with the STEMdiff-ACF attachment substrate (STEMCELL Technologies). Every 3–5 d, the cells were passaged, and 3 passages later, the cells were split in a 0.1% gelatin-coated 10 cm dish in MSC medium. The medium was refreshed every alternate day.

The differentiation potential of IMSCs was identified using the adipogenesis, osteogenesis and chondrogenesis differentiation kits (STEMCELL Technologies); iMSCs were seeded and cultured in differentiation medium for 2 to 3 w according to the manufacturer’s instructions. Further, they were stained with Oil Red O, Alizarin red or Alcian blue dye (Cyagen Biosciences Inc., Guangzhou, China), respectively.

Flow cytometric analysis of surface markers: A total of 5 × 10^4^ iMSCs in single form were washed and incubated in 100 µL 5% FBS with BB515-conjugated CD44, Precp-Cy5.5-conjugated CD73, PE-Cy7-conjugated anti-human CD90, APC-conjugated CD105, and BV421-conjugated anti-human CD34, CD45, and HLA-DR (BD Biosciences) at 4 °C for 30 min in the dark. The stained cells were washed with DPBS and analyzed using flow cytometry (CYTEK).

### 4.10. FVIII Assay Using Enzyme-Linked Immunosorbent Assay (ELISA)

Culture supernatants were harvested in triplicate from 12-well plates (iEPCs) or 6-well plates (iMSCs) 24 h after changing the medium. The cells were then dissociated and counted. After washing with DPBS, the cells were incubated in 500 µL sample dilution (Cedarlane, Burlington, ON, Canada) per 10^6^ cells for 20 min and then lysed using 3 freeze–thaw cycles. According to the manufacturer’s instructions, ELISA was performed using paired antibodies against ELISA-factor VIII:C (Cedarlane), according to the manufacturer’s instructions. In short, the labeled plate was coated with capture antibody for 2 h, and then the sample was added for incubating for 2 h. After incubation, detecting antibody was added. Finally, OD value for 490 nm was detected using the labeled instrument. The standard curves were constructed using serial dilutions of normal pooled plasma for 20, 10, 5, 2.5, 1.25, 0.625, 0.3125, 0.15625, and 0 ng/mL, with a correlation coefficient (R2) greater than 0.990 using a semilog fit.

### 4.11. Statistical Analysis

GraphPad Prism 8.0 was used for data analysis, *t*-test for two groups and one-way ANOVA in conjunction with Games-Howell’s or Dunn’s multiple comparisons test was used for more than two groups. All values were presented as the mean ± mean squared error (SEM). A *p* value < 0.05 was considered significant.

## 5. Conclusions

In summary, we identified an efficient enhancer element, C400, of the FVIII-Padua. The C400 extensively improved the transcription of *BDDF8* driven by human EF1α in a variety of cell types with different genetic backgrounds, and specifically increased the expression of FVIII protein in iEPCs. Our research provides a novel strategy to enhance expression of FVIII protein, facilitating the potential of clinical application in both viral and nonviral HA gene therapy.

## Data Availability

The data supporting the findings of this study are presented in this article including the Supplementary Materials and are available from the corresponding author upon reasonable request.

## Supplementary Materials

The following supporting information can be downloaded at: https://www.mdpi.com/article/10.3390/ijms25073635/s1.
