# Supplementary material for: Identification of the Efficient Enhancer Elements in FVIII-Padua for Gene Therapy Study of Hemophilia A
Source: Int J Mol Sci. 2024 Mar 24;25(7):3635. doi: 10.3390/ijms25073635 (PMC11011560; doi:10.3390/ijms25073635)
Supplement: Supplementary file 1 [file ijms-25-03635-s001.zip › ijms-2893420-supplementary.pdf]

Supplemental Information

Table S1. Sequences of the elements and promoters used in this study

Sequence name	Sequence (5’ → 3’)
C400	GCAGAGGGAGAAGCGCGCTGCCGGGTAGGCTCCAGGACAGCCTTGC<br>TGGATCTTACAGGGAGCTCTGGAGCTGGAATGAACCTTCCAGGTTGG<br>ACCAACATGTCCAGGCCTCTGTGTCCCTGCACTGAGCACTCATGAAG<br>TGCTGTCCATCCTGGGACAGGGCAGAGTCGGCGCTGGGAAGAGGCG<br>TGGTCTGCCCAGAGAGGGGGTGGGGCCTGTCCTGGAAATGGGCGGG<br>GCATGCCCTGGTAGAGGGGCGTGGTCTGTCTTGGGAAGGAAGGTGC<br>GTGGCCCCCACAGGCAACATTCCTCTGGGGCAATCCCGGCGCACACC<br>TCAGCGGAGGTGAAGGCTGACTTCCCACACCCAATGGCTGGGACTG<br>GGGAGTTCTTTCCTAGACTGCCATCCGGG
C175	AATGTTAAATGACTGAAAACTACATAGGCTTATGTTAAGACTAGTGCC<br>ATGGAAATTTTTAGAGGTAGATGACATTAGAGTATGTTTAAATATAAAT<br>TAATGTTCTTGCAGTGAGCCGAGATTGCGCCACTGCAGTCCGCAGTC<br>CGGCCTGGGCGACAGAGCGAGACTCCGTCTC
C927	TTGGTGAATGTGGAAGGGCATAATAATGTTAAATGACTGAAAACTACA<br>TAGGCTTATGTTAAGACTAGTGCCATGGAAATTTTTAGAGGTAGATGA<br>CATTAGAGTATGTTTAAATATAAATTAATGTTCTTGCAGTGAGCCGAGA<br>TTGCGCCACTGCAGTCCGCAGTCCGGCCTGGGCGACAGAGCGAGAC<br>TCCGTCTCAAAAAAAAAAAAAAAAAAATTAATGTTACTGGTTGTGTT<br>CCCTAGATGTAGACTCTGAGATGAAGATTAACATGCAGGAATTTTATT<br>TGGAGTGTTCTTGGGATCAACACCTGAGGAATCAGGGATAGGCAGAG<br>GGAGAAGCGCGCTGCCGGGTAGGCTCCAGGACAGCCTTGCTGGATC<br>TTACAGGGAGCTCTGGAGCTGGAATGAACCTTCCAGGTTGGACCAAC<br>ATGTCCAGGCCTCTGTGTCCCTGCACTGAGCACTCATGAAGTGCTGT<br>CCATCCTGGGACAGGGCAGAGTCGGCGCTGGGAAGAGGCGTGGTCT

	GCCCAGAGAGGGGGTGGGGCCTGTCCTGGAAATGGGCGGGGCATGC CCTGGTAGAGGGGCGTGGTCTGTCTTGGGAAGGAAGGTGCGTGGCC CCCACAGGCAACATTCCTCTGGGGCAATCCCGGCGCACACCTCAGCG GAGGTGAAGGCTGACTTCCCACACCCAATGGCTGGGACTGGGGAGT TCTTTCCTAGACTGCCATCCGGGCGCCCCTCACCCTCTTGCTGCTCAG CTCCAGGTCGTCGTGGGTTCAGGGCTCAGCTGCACGCTCCTGCCCGC GCCCTGGGCGTGATGGCACCCCCAGCCCCTGCCATTCTTCCCCCTCA CCCCCTCTCCCTGCCACTGCTCTGCATTGCCCTGGGTTAGCCTGGCGG GGCCAGGTGGCACCCGCCGTATACTCTTGCCTT
E546	TAGAATTTTTATTTTAATGAAACCCTTTTATACTTTAGATTATTTTGATG CTGATTAACAGGATAAGCTGACTCTGGACACATCCTGACAGCCTCTG CCAAGGTTTGAAATAAACAGCTGGGAAAATTCAGTTTTATATTATCTG TTCCCGCAGCTGCTCAGTTTCTTCTAACCACAGGTCAGGGAATATAAT AGGTTTTCCGATTAGTTTTAGATAAAATGTGAAAGAACAGTTCATGTC AAGGACAATGGTTGCCAAATATATTTGGCAGGATTTCTATATTGAATCC CAAAGGAAATACACAAACAAAACCCCACAAAAGTTAGGAAGGAGTA AAACCCAGGAACCCTGGAACATTTTGTCATTTACTATGCAGATTTGCC TGAAAGTGAGACAGGCAAATAAATCACATGTTTCTGCCAGCGTGGAA AATATTCACTCAAATGGCAAAGGTCTCAGGCTGGGGAGCTGGATATT GTCCTGTAATAGGTTCATCTCAGAACTGAATCACACACTTGGAGGGT GTTAATGCTCTTAGGAACATC
EF1α	GCTCCGGTGCCCGTCAGTGGGCAGAGCGCACATCGCCCACAGTCCCC GAGAAGTTGGGGGGAGGGGTCGGCAATTGAACCGGTGCCTAGAGAA GGTGGCGCGGGGTAAACTGGGAAAGTGATGTCGTGTACTGGCTCCGC CTTTTTCCCGAGGGTGGGGGAGAACCGTATATAAGTGCAGTAGTCGC CGTGAACGTTCTTTTTCGCAACGGGTTTGCCGCCAGAACACAGGTAA GTGCCGTGTGTGGTTCCCGCGGGCCTGGCCTCTTTACGGGTTATGGCC CTTGCGTGCCTTGAATTACTTCCACGCCCCTGGCTGCAGTACGTGATT CTTGATCCCGAGCTTCGGGTTGGAAGTGGGTGGGAGAGTTCGAGGC

	CTTGCGCTTAAGGAGCCCCTTCGCCTCGTGCTTGAGTTGAGGCCTGG CTTGGGCGCTGGGGCCGCCGCGTGCGAATCTGGTGGCACCTTCGCGC CTGTCTCGCTGCTTTCGATAAGTCTCTAGCCATTTAAAATTTTTGATGA CCTGCTGCGACGCTTTTTTTCTGGCAAGATAGTCTTGTAAATGCGGGC CAAGATCTGCACACTGGTATTTCGGTTTTTGGGGCCGCGGGCGGCGA CGGGGCCCGTGCGTCCCAGCGCACATGTTCGGCGAGGCGGGGCCTG CGAGCGCGGCCACCGAGAATCGGACGGGGGTAGTCTCAAGCTGGCC GGCCTGCTCTGGTGCCTGGCCTCGCGCCGCCGTGTATCGCCCCGCCC TGGGCGGCAAGGCTGGCCCGGTCGGCACCAGTTGCGTGAGCGGAAA GATGGCCGCTTCCCGGCCCTGCTGCAGGGAGCTCAAAATGGAGGAC GCGGCGCTCGGGAGAGCGGGCGGGTGAGTCACCCACACAAAGGAA AAGGGCCTTTCCGTCCTCAGCCGTCGCTTCATGTGACTCCACGGAGT ACCGGGCGCCGTCCAGGCACCTCGATTAGTTCTCGAGCTTTTGGAGT ACGTCGTCTTTAGGTTGGGGGGAGGGGTTTTATGCGATGGAGTTTCC CCACACTGAGTGGGTGGAGACTGAAGTTAGGCCAGCTTGGCACTTGA TGTAATTCTCCTTGGAATTTGCCCTTTTTGAGTTTGGATCTTGGTTCAT TCTCAAGCCTCAGACAGTGGTTCAAAGTTTTTTTCTTCCATTTCAGGT GTCGTGA
minP	TAGAGGGTATATAATGGAAGCTCGACTTCCAG
SV40	TGCATCTCAATTAGTCAGCAACCATAGTCCCGCCCCTAACTCCGCCCA TCCCGCCCCTAACTCCGCCCAGTTCCGCCCATTCTCCGCCCCATCGCT GACTAATTTTTTTTATTTATGCAGAGGCCGAGGCCGCCTCGGCCTCTG AGCTATTCCAGAAGTAGTGAGGAGGCTTTTTTGGAGGCCTAGGCTTT TGCAAA

Table S2. Primers used in the study

Name	Sequence (5'-3')
minP-PGL3-F	5'-TTACGCGTGCTAGCCCGGGCTCGAGTAGAGGGTATATAATGGAAGCTCGACTTCC-3'
minP-PGL3-R	5'-CCAACAGTACCGGAATGCCAAGCTTCTGGAAGTCGAGCTTCCATTATATACCCT-3'
EF1α-PGL3-F	5'-TTACGCGTGCTAGCCCGGGCTCGAGATGCGTGAGGCTCCGG-3'
EF1α-PGL3-R	5'-AGTACCGGAATGCCAAGCTTGGTTCACGACACCTGAAATGGA-3'
C400-PGL3-F	5'-AGAACATTTCTCTATCGATAGGTACCGCAGAGGGAGAAGCGCGCTG-3'
C400-PGL3-R	5'-GCCCGGGCTAGCACGCGTAACCCGGATGGCAGTCTAGGAA-3'
C575-PGL3-F	5'-AGAACATTTCTCTATCGATAGGTACCAATGTTAAATGACTGAAAACTACATAGGCT-3'
C175-C400-R	5'-CAGCGCGCTTCTCCCTCTGCGAGACGGAGTCTCGCTCT-3'
C175-C400-F	5'-AGAGCGAGACTCCGTCTCGCAGAGGGAGAAGCGCGCTG-3'
E5-PGL3-F	5'-AGAACATTTCTCTATCGATAGGTACCTAGAATTTTTATTTTAATGA-3'
C-E-R	5'-CAGCGCGCTTCTCCCTCTGCGATGTTCCTAAGAGCATTAA-3'
C-E-F	5'-TTAATGCTCTTAGGAACATCGCAGAGGGAGAAGCGCGCTG-3'
C4-tandem-R	5'-CAGCGCGCTTCTCCCTCTGCCCCGGATGGCAGTCTAGGAA-3'
C4-tandem-F	5'-TTCCTAGACTGCCATCCGGGGCAGAGGGAGAAGCGCGCTG-3'
C927-PGL3-F	5'-AGAACATTTCTCTATCGATAGGTACCTTGGTGAATGTGGAAGGGCA-3'
C927-PGL3-R	5'-GCCCGGGCTAGCACGCGTAAAAGGCAAGAGTATACGGCGG-3'
BDDF8-BQ-F	5'-TGTTGGAGGCTTGGAACTCT-3'
BDDF8-BQ-R	5'-TTTACTTCCTCTAGTGCTAGTCGCGACGTACGATGGAAATAGAGCTCTCCACCTGC-3'
EF1α-*BDDF8*-F	5'-CTCTAGTGCTAGCGTACGTCGCGAATGCGTGAGGCTCCGG-3'
EF1α-*BDDF8*-R	5'-GGTGGAGAGCTCTATTTcCATTCGGGTTCACGACACCTGAAATGGA-3'

BDDF8-1896-R	5'-GATGTTGGAGGCTTGGAACTCT-3'
BDDF8-0-F	5'-ATGgAAATAGAGCTCTCCACCTGC-3'
BDDF8-1833-F	5'-CGCTTTCTCCCCAATCCAGC-3'
BDDF8-3646-R	5'-CCAACAGATCCACCTTGATCCAAG-3'
EF1α-pro-F	5'-TCAAGCCTCAGACAGTGGTTC-3'
EF1α-R	5'-CCCCAAAAAACCGAAATACCAGTG-3'
BGH-R	5'-TAGAAGGCACAGTCGAGG-3'
SP6-F	5'-ATTTAGGTGACACTATAGA-3'
C4 *F8*-F	5'-TACTTCCTCTAGTGCTAGCGTACGCCCGGATGGCAGTCTAGGAA-3'
C4 *F8*-R	5'-GAGCCTCACGCATTCGCGAGCAGAGGGAGAAGCGCGCTG-3'
C9 *F8*-F	5′-TACTTCCTCTAGTGCTAGCGTACGTTGGTGAATGTGGAAGGGCA-3′
C9 *F8*-R	5′-GAGCCTCACGCATTCGCGAAAGGCAAGAGTATACGGCGG-3′
qPCR-*GAPDH*-F	5′-GGGGAGCCAAAAGGGTCATCATCT-3′
qPCR-*GAPDH*-R	5′-GACGCCTGCTTCACCACCTTCTTG-3′
F8 RT-19F	5'-GCTGGGATGAGCACACTTTT-3'
F8 RT-23R	5'-TCAACTCCATGCGAAGAGTG-3'
F8 RT-23F	5'-CACTCTTCGCATGGAGTTGA-3'
F8 RT-26R	5'-GGGGGTGAATTCGAAGGTAGC-3'
rDNA-screen-F	5'-CCTGAGAAACGGCTACCACA-3'
rDNA-screen-R	5'-GAACTGCTTCCTTCACGACAT-3'
Southern probe-F	5'-GCCGAGAAAGTATCCATCA-3'
Southern probe-R	5'-CAGAGTCCCGCTCAGAAG-3'

Table S3. The three candidate off-target loci of left TALEN in the genome

Off-target site	TALEN recognition sites	Mismatch, bp	Chromosome	Closest gene
Off-target-1	TAGAGAAGACGG	1	5	N/A
Off-target-2	GTAT-GACTGTCTTCTC	3	9	CACNA1B
Off-target-3	TGAGAAAACG	1	2	CCDC93

The top three potential off-target sites of TALEN-L predicted by PROGNOS website (http://bao.rice.edu/bao/Research/BioinformaticTools/prognos.html). The mismatch bases are indicated in red colore.

Figure S1. Cluster of transcription factor (TF) binding sites in C927 predicted by Cister.

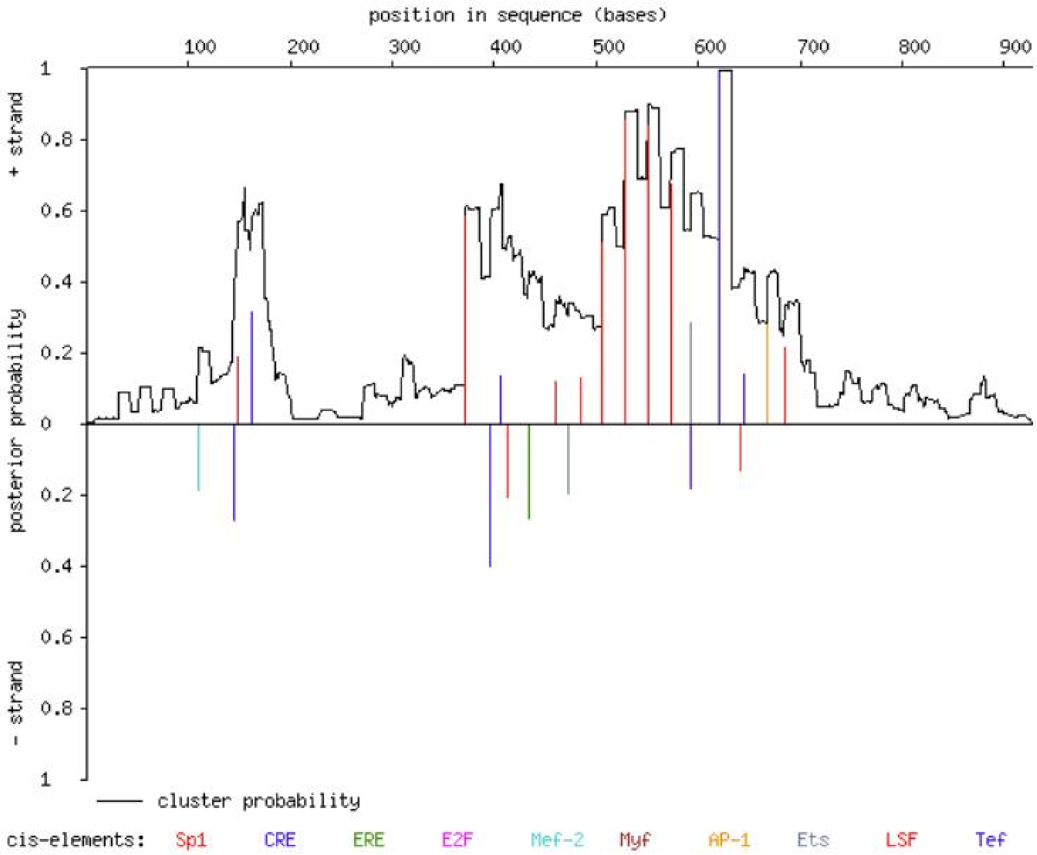

The black peaks indicate the overall probability of cis elements cluster binding to TFs, and the vertical coloured lines indicate the probability of the TF binding to that particular location. [1]

Figure S2. Sequencing of luciferase gene reporter plasmids

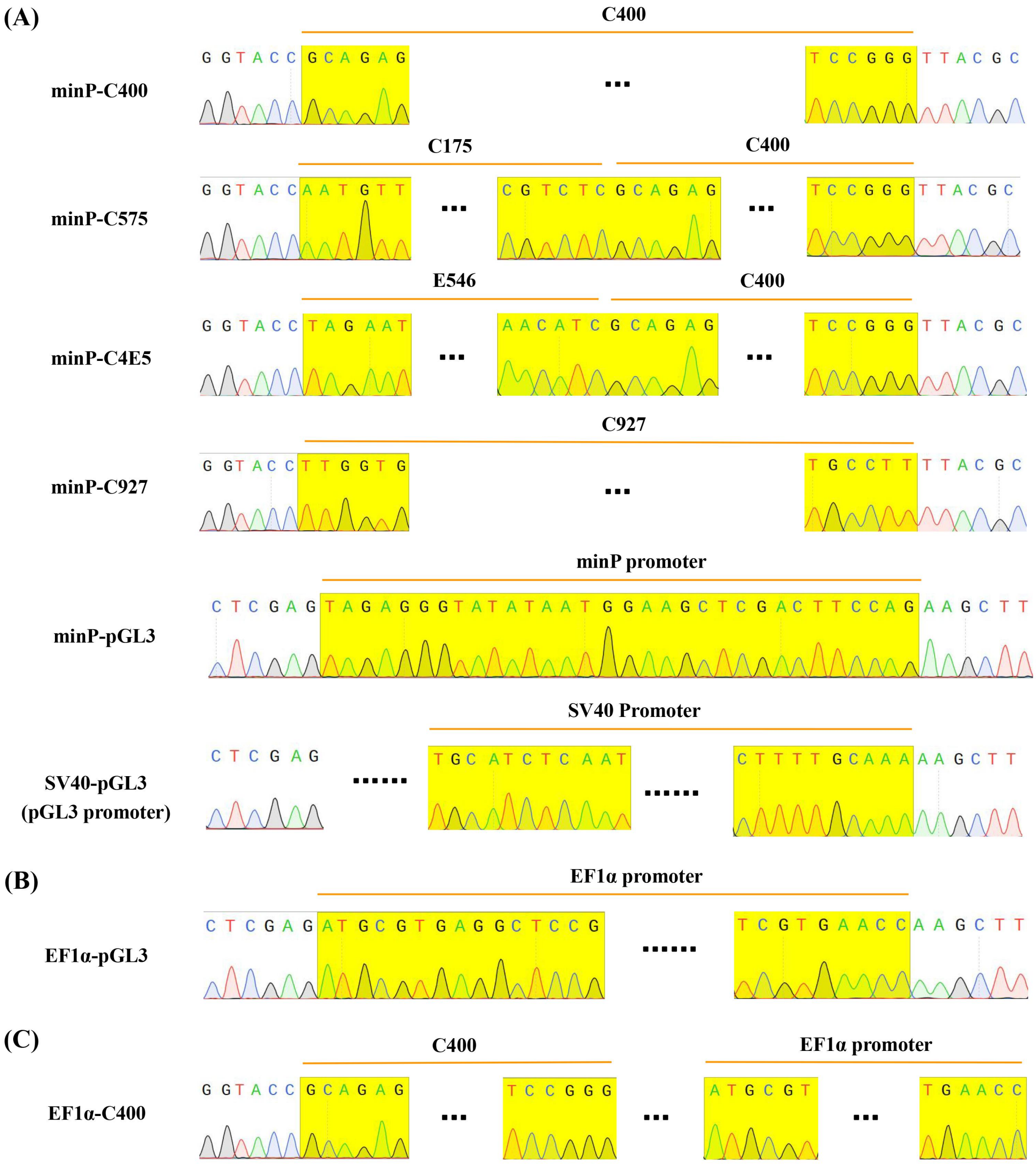

(A) Sequencing of minP-luciferase plasmids with candidate enhancer elements in FVIII-Padua inserted upstream the minP promoter, the element or promoter completely consistent with the theoretical sequences were in yellow; sequencing of SV40-pGL3 (pGL3-Promoter Vector) was shown below, the SV40 promoter is 10 bp (5' -ATCTGCGATC-3') away from the restriction site. (B) Sequencing of EF1α-pGL3, the EF1α promoter completely consistent with the theoretical sequence was in yellow. (C) Sequencing of EF1α-C400, the efficient enhancer C400 was inserted upstream the EF1α promoter, both the enhancer C400 and the promoter EF1α were completely

match the theoretical sequences (displaying in yellow).

(Showing the junctions of the pGL3 vector backbones and the elements or promoters, and the pGL3 vector backbone were in white. The peaks in each color represent specific bases, A: green, T: red, C: blue, G: black.)

Figure S3. Identification of rDNA-specific integrated iPSCs

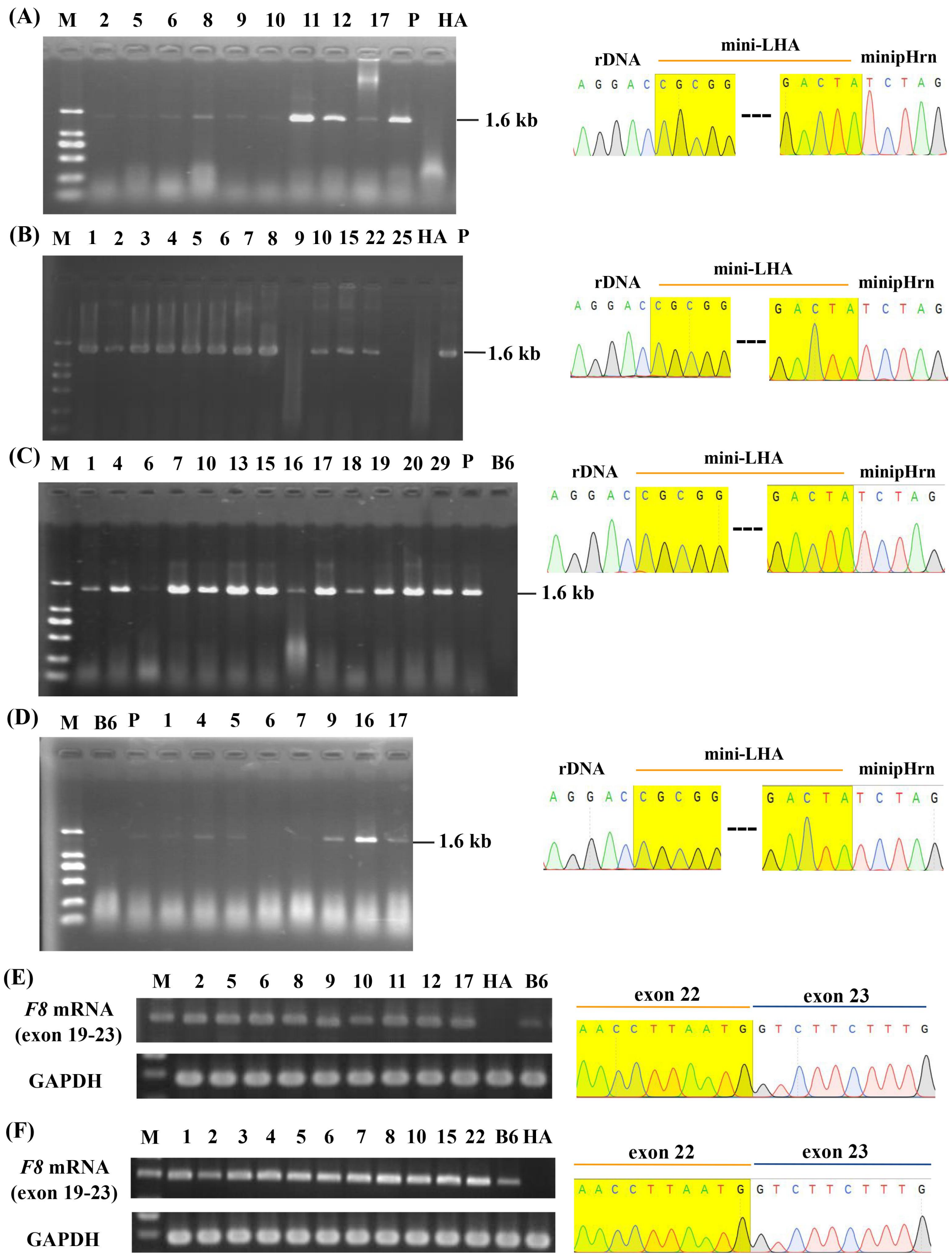

(A) Monoclonal identification of HA-iPSCs nucleofected with minipHrn-EF1α-BDDF8. Amplified by primers spanning LHA, all identified clones were positive for a 1.6 kb band (left), and the sequencing results of the products were also consistent with the theoretical sequences

(right). (B) Monoclonal identification of HA-iPSCs nucleofected with minipHrn-C400-EF1 α -BDDF8. Except for clone 9 and 25, all other identified clones were positive. (C) Monoclonal identification of B6-iPSCs nucleofected with minipHrn-EF1 α -BDDF8, all identified clones were positive. (D) Monoclonal identification of B6-iPSCs nucleofected with minipHrn-C400-EF1 α -BDDF8. Except for clone 6, all other identified clones were positive. (Left: M, DL2000 DNA ladder; P, Positive control). (Right: Yellow is the sequence of LHA, showing the connection between LHA and rDNA region or the backbone of minipHrn vector). (E), (F) Identification and sequencing of *F8* transcripts (exon19-23) in HAF8-iPSCs and HAC4-iPSCs. HA is a negative control, and B6 is a positive control. (Left: M, DL2000 DNA ladder; Right: showing the junction of exon 22 and exon 23).

(The peaks in each color represent specific bases, A: green, T: red, C: blue, G: black.)

Figure S4. Security identification of rDNA-specific integrated HA-iPSCs

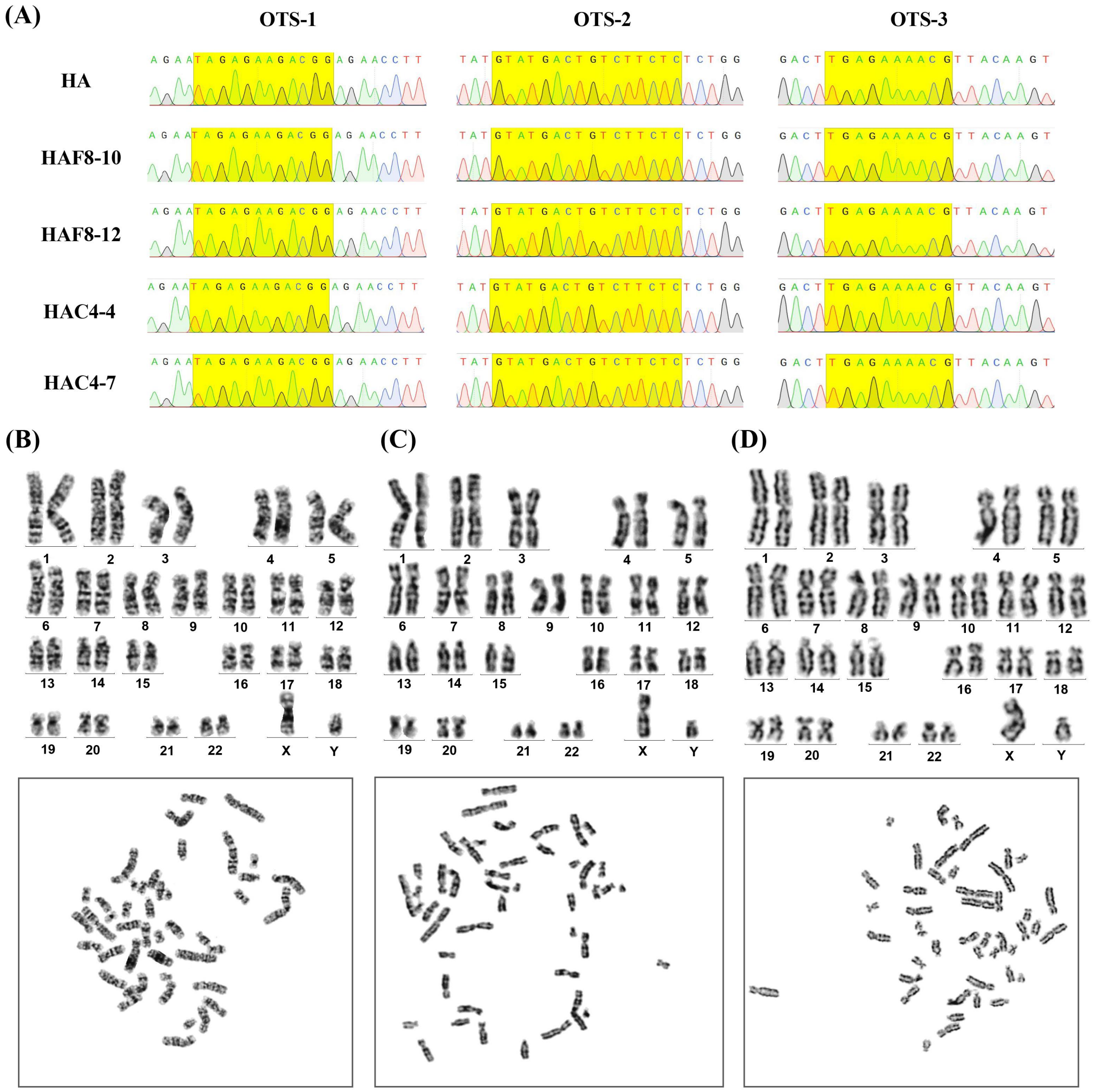

(A) The Sanger sequencing of the predicted off-target sites (OTS-1/2/3), and the unmodified HA-iPSCs as the control, the sequences of the recognition sites (in yellow) were consistent with those before targeting. The peaks in each color represent specific bases, A: green, T: red, C: blue, G: black. (B), (C), (D) Respectively correspond to the karyotype of HA-iPSCs, HAF8-10, and HAC4-4, showing the karyotype analysis (above) and the original image of karyotype collection (below).

Figure S5. Security identification of rDNA-specific integrated B6-iPSCs

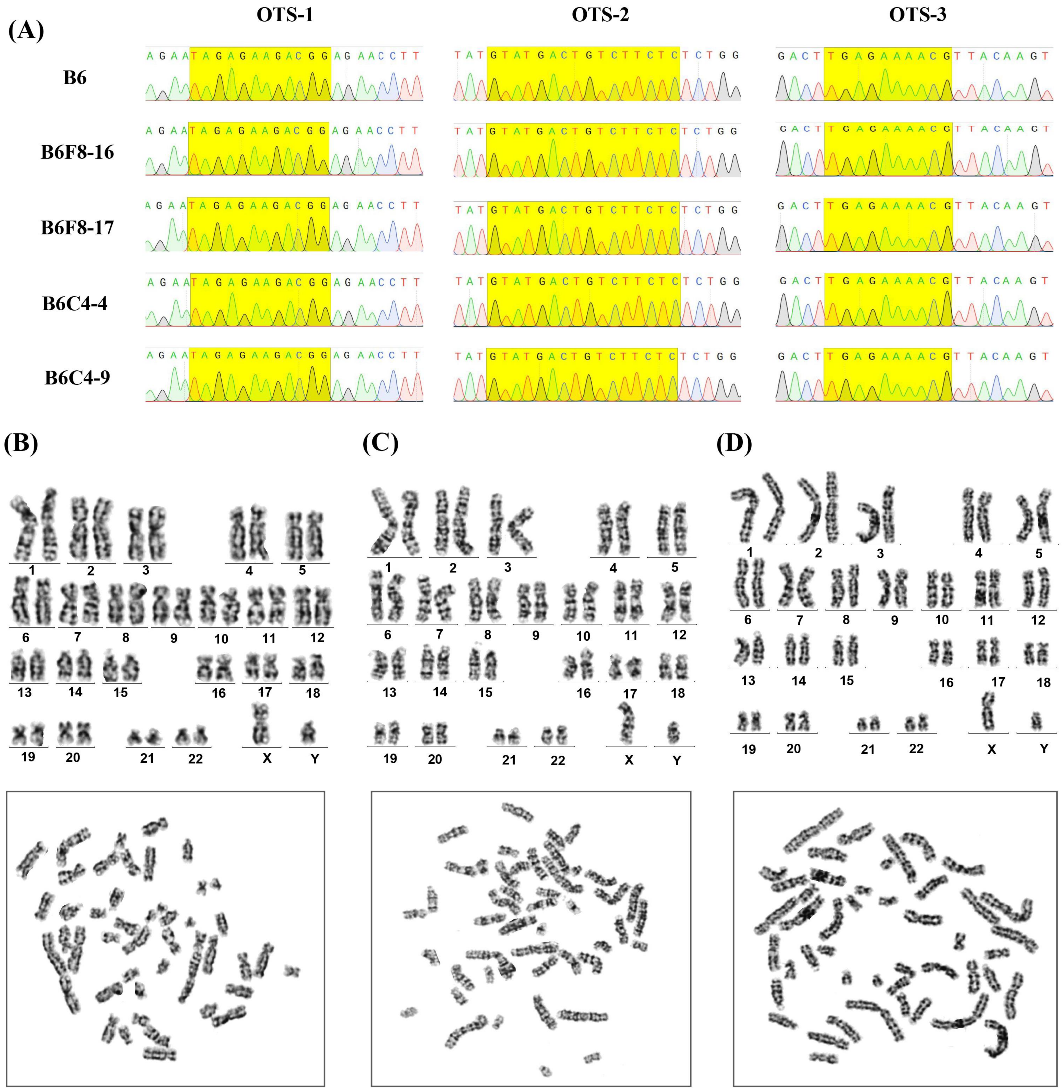

(A) The Sanger sequencing of the predicted off-target sites (OTS-1/2/3), and the unmodified B6-iPSCs as the control, the sequences of the recognition sites (in yellow) were consistent with those before targeting. The peaks in each color represent specific bases, A: green, T: red, C: blue, G: black. (B), (C), (D) Respectively correspond to the karyotype of HA-iPSCs, B6F8-16, and B6C4-9, showing the karyotype analysis (above) and the original image of karyotype collection (below).

Figure S6. Identification for the pluripotential of rDNA-specific integrated iPSCs

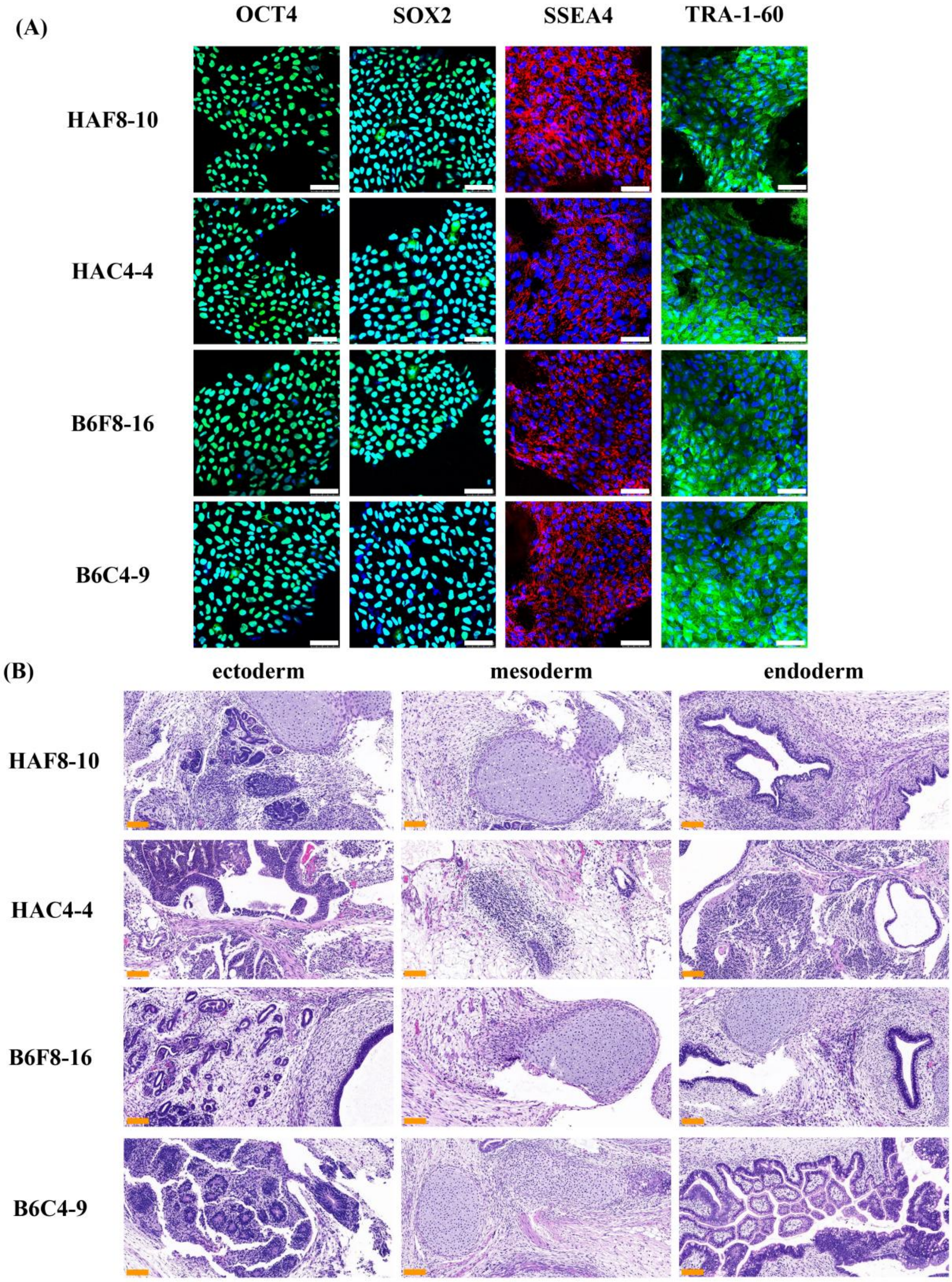

(A) Immunofluorescence of stemness markers, OCT4, SOX2, and TRA-1-60 were labeled with green, SSEA4 was labeled with red, and DAPI (blue) was used to stain the nucleus. Scale bar: 75 μm. (B) Three germ layer differentiation identification, H&E staining of teratoma, including three

germ layers: ectoderm (nervous tissue), mesoderm (cartilage) and endoderm (respiratory epithelium). The images of ectoderm and mesoderm of HAF8-10 were partially overlapped. Scale bar: 100 μm.

Figure S7. Flow cytometry analysis of iMSCs

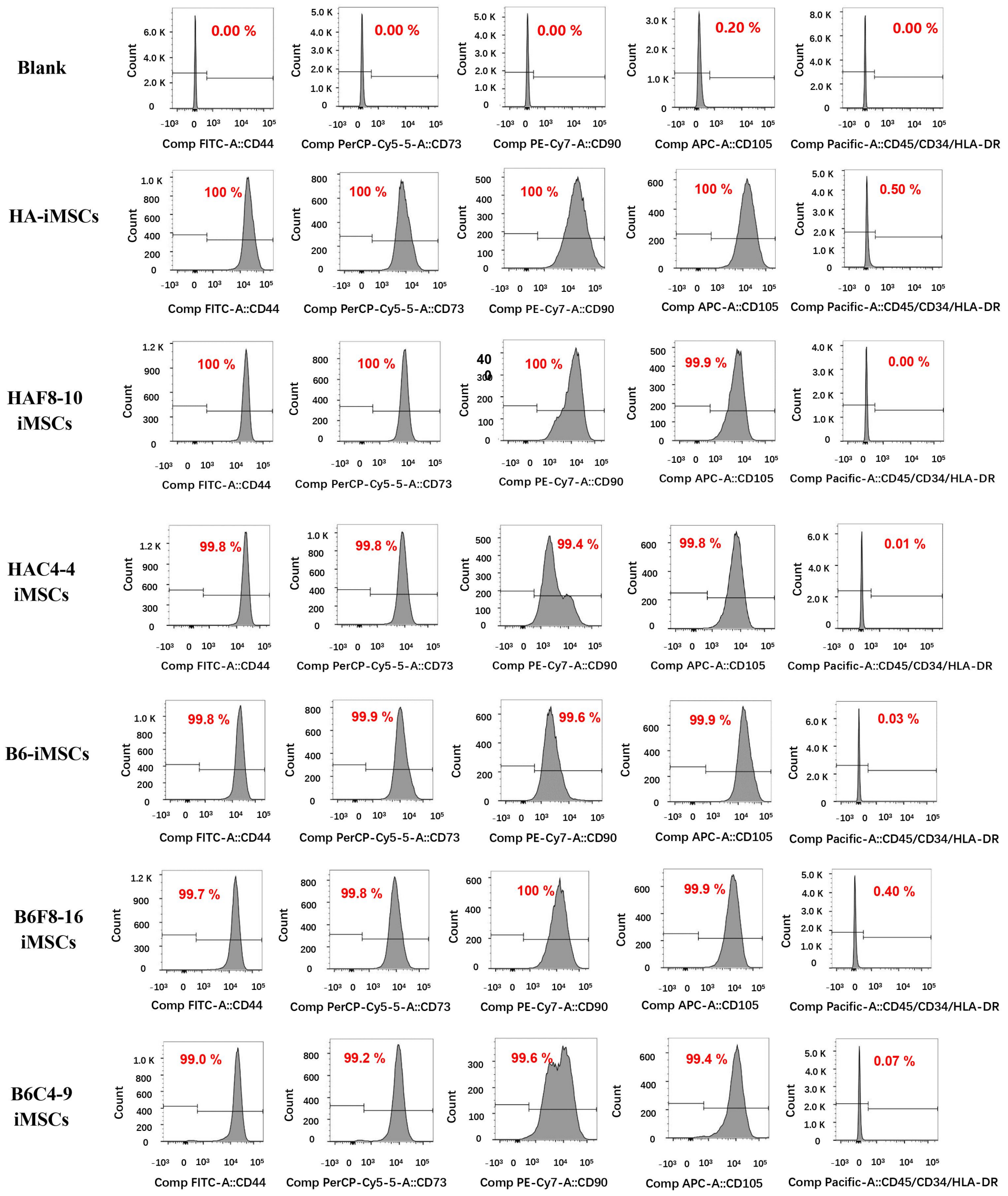

The expression of cell surface markers CD44, CD73, CD90, CD105, CD34, CD45 and HLA-DR was detected by flow cytometry, and the cell ratio was marked in %.

Figure S8. Identifification of adipogenic, chondrogenic, and osteogenic differential potential of iMSCs

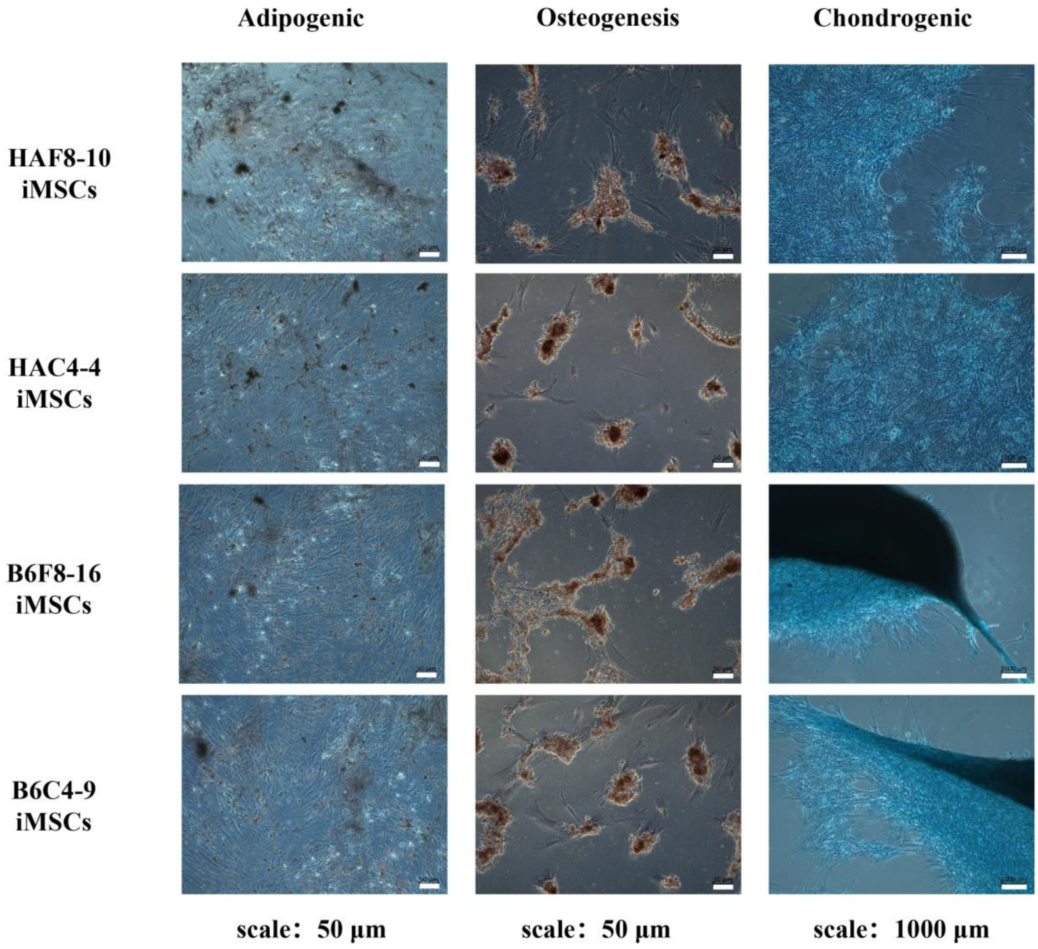

The staining of adipose, osteoblast, and chondrocytes differentiated from rDNA-specific integrated iMSCs, and the scale bars were 50 μm, 50 μm, and 1000 μm, respectively.

references

1. Simioni, P.; Cagnin, S.; Sartorello, F.; Sales, G.; Pagani, L.; Bulato, C.; Gavasso, S.; Nuzzo, F.; Chemello, F.; Radu, C. M.; Tormene, D.; Spiezia, L.; Hackeng, T. M.; Campello, E.; Castoldi, E., Partial F8 gene duplication (factor VIII Padua) associated with high factor VIII levels and familial thrombophilia. *Blood* **2021,** 137, (17), 2383-2393.
